# The efficacy of therapies for post-stroke depression in aging: An umbrella review

**DOI:** 10.3389/fnagi.2022.993250

**Published:** 2022-08-23

**Authors:** Jinlu Xie, Xiwen Geng, Fangcheng Fan, Xuyan Fu, Shuaibing He, Tao Li

**Affiliations:** ^1^Key Laboratory of Vector Biology and Pathogen Control of Zhejiang, School of Medicine, Huzhou Central Hospital, Huzhou University, Huzhou, China; ^2^Experimental Centre, Shandong University of Traditional Chinese Medicine, Jinan, China; ^3^School of Pharmacy, Minzu University of China, Beijing, China

**Keywords:** post-stroke depression, meta-analysis, systematic review, umbrella review, therapies

## Abstract

Post-stroke depression (PSD) is a common complication after stroke. PSD is associated with emotional disorders and psychological dependence, which are potential risk factors for stroke recurrence and suicidality. This study aimed to perform an umbrella review of therapies for PSD through a comprehensive literature search. A systematic search was conducted in the PubMed and Web of Science by two independent authors. We examined the Hamilton Depression Scale (HAMD), Activities of daily living (ADL), Neurologic function as efficacy endpoints, and the incidence of adverse events as safety profiles. Seventeen eligible studies, including 267 clinical trials were included in this study. The results showed that High-Frequency Repetitive Transcranial Magnetic Stimulation (HfrTMS), Acupuncture/EA+conventional treatment, Escitalopram, Modified Sini San, Moxibustion, Xiaoyao Formula, Paroxetine, Chinese herbal medicine, Exercise, Citalopram, and Cognitive behavioral therapy are beneficial for improving the depression symptoms of patients with PSD. HfrTMS and Sertraline may have an impact on slowing the scores of activities of daily living or neurologic function. In addition, Acupuncture/EA+conventional, Escitalopram, Citalopram, Sertraline, and Fluoxetine showed no serious adverse events in PSD patients. Our study demonstrated that 11 treatment methods can effectively improve the condition of PSD patients.

## Introduction

Stroke is a devastating disease causing significant neurologic disability, the second leading cause of death, and a major cause of long-term disability worldwide (Khoshnam et al., 2017). Post-stroke depression (PSD) is a common complication after stroke. It afflicts around 33% of stroke survivors and affects mortality rate, rehabilitation outcomes, and quality of life (Paolucci, 2017). In addition, emotional disorders and psychological dependence are potential risk factors for stroke recurrence and suicidality (Speranza et al., 2004).

The onset of PSD depends on psychosocial factors, the severity of the general vascular injury, stroke, and disability, and the interaction of other disorders (Göthe et al., 2012). The pathophysiology of PSD is closely related to vascular injury. There are many notable differences between PSD and major depression disorder (MDD): PSD has more serious depressive symptoms than MDD (Loubinoux et al., 2012); In addition, PSD shows more cognitive impairment and less sleep/circulation disturbance compared with MDD; The patients with PSD have a high prevalence of physical disabilities, such as aphasia, sensory loss, and motor/gait disorders (Cumming et al., 2010; Medeiros et al., 2020). In most cases, PSD occurs in the first month after the onset of stroke, tends to be chronic over time, and interferes with functional recovery (Arcadi et al., 2021). PSD limits participation in rehabilitation, reducing physical, social, and cognitive functions, and impairing neuroplasticity (Kutlubaev and Hackett, 2014). In addition, PSD increases the risk of mortality after stroke and recurrent stroke. The reduction of motivation-related PSD is considered to lead to the reduction of the motivation related to PSD, which in turn, leads to the reduction of the patient's willingness to adhere to preventive treatment (Miranda et al., 2018).

PSD is a common psychological complication of stroke, affecting about one-third of patients after stroke, harming the morbidity and mortality rates of patients.Therefore, it is important to develop effective post-stroke identification and treatment strategies. In addition, PSD is a common and complex psychiatric disease that delays the recovery of rehabilitation function and increases cognitive impairment. The etiology of PSD appears to be originated from physical and psychosocial stress, alone or in combination (Li et al., 2014). Furthermore, PSD negatively affects functional outcomes (Robinson and Jorge, 2016). The mechanism of PSD interferes with or affects functional recovery is considered to be the reduction of motivation and cognitive abilities (Vataja et al., 2001). There are several treatment strategies for PSD, including pharmacological and psychotherapy (Nabavi et al., 2014).

The clinical treatment of PSD is not exactly the same as MDD, which needs to be specifically approached. Pharmacotherapy and psychotherapy have traditionally been used in the treatment of PSD. Selective serotonin reuptake inhibitors (SSRIs) and serotonin-norepinephrine reuptake inhibitors (SNRIs) are frequently selected for the treatment of PSD. It is worth noting that SSRIs are first-choice drugs (Mortensen and Andersen, 2015), but many patients experience many adverse events with the treatment, such as the risk of bone fractures and nausea (Richter et al., 2021). Psychotherapy is commonly used in the treatment of PSD patients. Psychotherapy includes hfrTMS, problem-solving treatment, meridian acupoint massage, music therapy, exercise, motivational interview, and robot-assisted neural rehabilitation (Hadidi et al., 2017). The existing evidence shows that drug intervention and psychotherapy can prevent depression and improve the mood of PSD (Zhang et al., 2021c). However, the hfrTMS may increase the risk of seizures, and other psychotherapies are still inconclusive therapies for PSD (Wang et al., 2018; Frey et al., 2020). In addition, patients with PSD also consider seeking Traditional Chinese medicine (TCM) therapy as treatment, such as acupuncture (Hung et al., 2019), Chinese herbal medicines (Zhang et al., 2021a), and moxibustion (Guo et al., 2022). Increasing studies have been conducted to evaluate the therapeutic effect of TCM therapy on the treatment of PSD (Huang et al., 2018).

This study aims to provide an umbrella review of PSD therapies through a comprehensive literature search and to reach a clear conclusion by integrating the available meta-analyses and systematic reviews to identify an efficacious treatment for PSD patients that are commercially available.

## Methods

Our study was performed under the Preferred Reporting Items for Systematic Reviews and Meta-analyses (PRISMA) statement (Moher et al., 2009).

### Search strategy and quality assessment

Two independent authors conducted a preliminary screening in the PubMed and Web of Science to search for articles that contained search terms most related to our main construct of interest. The search terms were: (post-stroke depression) and (systematic review or meta-analysis). Meta-analyzed and systematic reviews of treatment methods for PSD patients were included in this study. Inclusion criteria were: (1) published in peer-reviewed journals until May 2022; (2) English-language literature; (3) published meta-analyses or systematic reviews; (4) participants in the general population. The studies were excluded if (1) duplicates; (2) unpublished studies; (3) not published in English; or (4) studies reported insufficient details and outcomes.

The meta-analyses and systematic reviews were evaluated using the AMSTAR tool (Shea et al., 2007). Studies were graded as low, medium, and high quality with an AMSTAR score of 0–4, 5–8, and 9–11, respectively.

### Data extraction

The main characteristics of the selected studies were extracted in a table, including the first authors, publication year, number of studies, regimens for the treatment, and main outcomes. We included results with at least one of the assessment scales: (1) Hamilton Depression Scale (HAMD), (2) Activities of daily living (ADL), (3) Neurologic function, and (4) incidence of adverse events. Data extracted from studies include the number of studies, the number of patients, standardized mean difference/mean difference or Risk Ratio/odds ratio, and heterogeneity (I^2^).

### Statistical analysis

The sample size and standardized mean difference/mean difference were calculated on the assessment scales. HAMD was used to provide an assessment indicator of depression and evaluate the effectiveness of the antidepressants for PSD. The NIHSS and ADL scales were performed to evaluate the neurologic function after stroke. The incidence of adverse events was assessed, and the Risk Ratio/ odds ratio was calculated. The selection of assessments was extracted on study size, sample size, standardized mean difference (SMD) /mean difference (MD) or relative ratio (RR) /odds ratio (OR), and heterogeneity (I^2^). The percentages of 0–25%, 26–50%, and 51–75% were classified as mild, moderate, and significant. If I^2^ > 50%, a random-effects model was used for the analysis, or the data was analyzed on the fixed-effects model.

## Results

### Literature search and study selection

**Figure 1:**
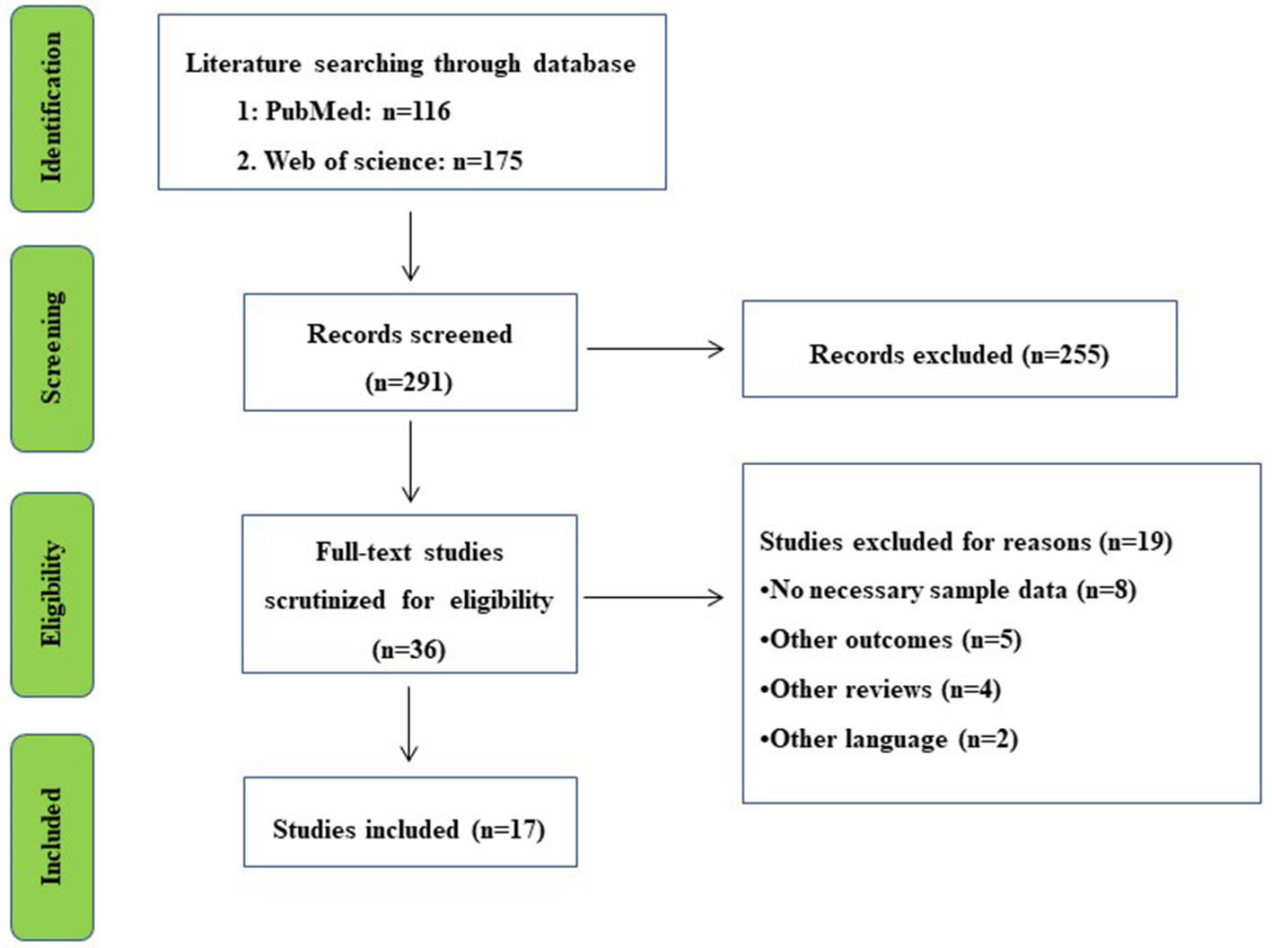
Literature review flowchart.

Overall, a total of 291 records were identified from PubMed and Web of Science databases. Titles and abstracts were screened for full-text scrutiny. In all, 19 studies were excluded due to the following reasons: No necessary sample data (*n* = 8), Other outcomes (*n* = 5), Other reviews (*n* = 4), and Other languages (*n* = 2) (Figure 1). Thus, 17 studies were included in the umbrella review (Yi et al., 2010; Eng and Reime, 2014; Ren et al., 2015; Tan et al., 2015; Feng et al., 2018, 2022; Jin et al., 2018; Wang et al., 2018, 2021; Liu et al., 2019, 2021; Li et al., 2020; Cai et al., 2021; Lee et al., 2021; Zhang et al., 2021a,b; Guo et al., 2022). Study characteristics and quality ratings of 17 meta-analyses/systematic reviews were summarized in Table 1.

**Table 1:** Description of included studies.

**References**	**Conditions**	**Studies** **Included**	**Study** **duration**	**Type of** **included** **studies**	**Outcomes**	**AMSTAR** **scores**	**Study quality**
Liu et al., 2019	High-Frequency Repetitive Transcranial Magnetic Stimulation	17	2-12W	RCTs	HAMD, BI or Modified Barthel Index, adverse events	9/11	High
Liu et al., 2021	Acupuncture	17	4W-6M	RCTs	HAMD	8/11	High
Feng et al., 2022	Escitalopram	11	4W-12M	RCTs	HAMD, Incidence of PSD, NIHSS, FM, Adverse events	9/11	High
Wang et al., 2021	Electroacupuncture	19	4-12W	RCTs	HAMD, Adverse events	9/11	Medium
Cai et al., 2021	Modified sini san	7	8W	RCTs	HAMD, Adverse events	10/11	High
Guo et al., 2022	Moxibustion	14	3-12W	RCTs	HAMD, MESSS, effective rate	9/11	High
Jin et al., 2018	XIAOYAO FORMULA	7	2-8W	RCTs	HAMD, Adverse events	7/11	High
Li et al., 2020	Paroxetine	4	4-12W	RCTs	HAMD	9/11	High
Feng et al., 2018	Sertraline	11	6-52W	RCTs	HAMD, Incidence of PSD, NIHSS, BI, Adverse events	9/11	High
Zhang et al., 2021a	Chinese herbal medicines	18	4-12W	RCTs	HAMD, SSS	10/11	High
Eng and Reime, 2014	Exercise	13	4-13W	RCTs	HAMD, GDS, CES	8/11	High
Lee et al., 2021	Non-Pharmacological Interventions	13	12-16W	RCTs	HAMD	8/11	High
Ren et al., 2015	Herbal medicine	26	1-8W	RCTs	HAMD	9/11	High
Tan et al., 2015	Citalopram	48	2-12W	RCTs	HAMD	10/11	High
Wang et al., 2018	Cognitive behavioral therapy	23	2-40W	RCTs	HAMD, SDS	10/11	High
Yi et al., 2010	Fluoxetine for the prophylaxis	6	4-12W	RCTs	HAMD, Adverse events	9/11	High
Zhang et al., 2021b	Acupuncture combined with antidepressants	13	4-8W	RCTs	HAMD, NIHSS, BI	9/11	High

### Depression rating scores

From our search, all 17 studies included the depression rating scores of the treatments in the HAMD score (Table 2). High-Frequency Repetitive Transcranial Magnetic Stimulation (hfrTMS; SMD: −1.01, 95%CI −1.36 – −0.66), Acupuncture/EA+conventional treatment (SMD: −2.72, 95%CI −3.61 – −1.82), Escitalopram (SMD: −1.15, 95%CI −2.21 – −0.09), Modified Sini San (MD: −6.68, 95%CI −9.22 – −4.14), Moxibustion (MD: −1.17, 95%CI −1.55 – −0.79), Xiaoyao Formula (MD: −5.21, 95%CI −7.48 – −2.95), Paroxetine (MD: −9.79, 95%CI −16.94 – −2.64), Chinese herbal medicine (MD: −3.17, 95%CI −4.12 – −2.22), Exercise (SMD: −0.13, 95%CI −0.26 – −0.01), Citalopram (MD: −0.43, 95%CI −0.85 – −0.01), Cognitive behavioral therapy (SMD: −0.76, 95%CI −1.22 – −0.29), showed better outcomes compared to the controls that received antidepressants such as other SSRIs, TCMs, TCAs, and placebo.

**Table 2:** Results of pairwise meta-analyses for HAMD.

**Pairwise meta-analyses**								
**Comparative** **medications**	**Reference** **medications**	**Number of studies**	**Number of patients**	**SMD**	**MD**	**95% CI**	**I^2^**	***P*-value**
High-Frequency Repetitive Transcranial Magnetic Stimulation	Control	15	1053	−1.01		[−1.36, −0.66]	85	*P* < 0.00001
Acupuncture/EA+ conventional	Conventional treatment	2	157	−2.72		[−3.61, −1.82]	0	*P* < 0.00001
Acupuncture/EA (+placebo)	Antidepressants (+sham acupuncture)	4	494	−1.55		[−4.36, 1.26]	95	*P* ≤ 0.28
Escitalopram	Control	4	320	−1.15		[−2.21, −0.09]	95	*P* < 0.00001
Electroacupuncture	Antidepressants	19	1572	−0.04		[ −0.16, 0.07]	0	*P* = 0.69
Modified sini san	Control	6	468		−6.68	[ −9.22, −4.14]	97	P < 0.00001
Moxibustion	Control	14	863	−1.17		[−1.55, −0.79]	86	*P* < 0.00001
Xiaoyao Formula	Antidepressants	7	607		−5.21	[−7.48, −2.95]	97	*P* < 0.00001
Paroxetine	Control	2	98		−9.79	[−16.94, −2.64]	95	*P* = 0.007
Chinese herbal medicine	Control	15	1367		−3.17	[ −4.12, −2.22]	87	*P* < 0.001
Exercise	Control	13	1022	−0.13		[ −0.26, −0.01]	6	*P* = 0.03
Citalopram	Other SSRIs	9	624	−0.43		[−0.85, −0.01]	85	*P* = 0.00
Citalopram	TCAs	12	700	−0.31		[−0.50, −0.12]	35	*P* = 0.11
Citalopram	TCMs	4	285	0.22		[ −0.20, −0.65]	69	P=0.02
No antidepressants	Control	7	859	−0.76		[−1.22, −0.29]	91	*P* = 0.001
Use of antidepressants	Control	14	970	−0.95		[−1.20, −0.71]	69	*P* < 0.00001
Some patients used antidepressants	Control	2	143	−0.2		[−0.53, 0.13]	0	*P* = 0.24
Fluoxetine	Control	4	217		−3.97	[−9.85, 1.90]	97	*P* = 0.19
Manual acupuncture +antidepressants	Control	6	483		−3.54	[−4.54, −2.55]	60	*P* < 0.00001
Electroacupuncture +antidepressants	Control	7	421		−3.66	[−4.58, −2.74]	28	*P* < 0.00001

### Activities of daily living

Table 3 presents the results of the effects of the treatments on activities of daily living. ADL scores assessed; only 2 studies. The results revealed that hfrTMS (MD: 1.09, 95% CI: 0.34–1.84) and Sertraline (MD: 11.48, 95% CI: 4.18–18.78) led to greater improvement than control group.

**Table 3:** Results of pairwise meta-analyses for ADL.

		**Pairwise meta-analyses**
**Comparative medications**	**Reference medications**	**Number of studies**	**Number of patients**	**MD**	**95% CI**	**I** ^**2**^	***P*** **-value**
High-Frequency Repetitive	Control	3	313	1.09	[0.34, 1.84]	89	*P* = 0.004
Transcranial Magnetic							
Stimulation							
Sertraline	Control	7	1032	11.48	[ 4.18, 18.78]	97	*P* = 0.002

### Neurologic function

The effects of the treatments on neurologic function were assessed by NIHSS (Table 4). Patients administered with hfrTMS (MD: −0.91, 95% CI: −1.19 – −0.63), Sertraline (MD: −3.44, 95% CI: −6.66 – −0.21), and Fluoxetine (MD: −4.72, 95% CI: −8.31 – −1.13) showed better neurologic function than the control.

**Table 4:** Results of pairwise meta-analyses for neurologic function.

**Pairwise meta-analyses**							
**Comparative** **medications**	**Reference** **medications**	**Number of studies**	**Number of patients**	**MD**	**95% CI**	**I^2^**	***P*-value**
High-Frequency Repetitive	Control	4	221	−0.91	[−1.19, −0.63]	0	*P* < 0.00001
Transcranial Magnetic							
Stimulation							
Sertraline	Control	4	328	−3.44	[−6.66, −0.21]	82	*P* = 0.04
Fluoxetine	Control	2	157	−4.72	[−8.31, −1.13]	82	*P* = 0.01

### Adverse events

The studies of patients with adverse events performed a beneficial effect of Acupuncture/EA(+placebo) (RR: 0.16, 95% CI: 0.07–0.39), Electroacupuncture (RR: 0.21, 95% CI: 0.14–0.32), Modified Sini San (OR = 0.12, 95% CI: 0.06–0.24), and Chinese herbal medicine (RR: 0.49, 95% CI: 0.31–0.77) treatment compared to control group. In addition, there were no significant differences between Acupuncture/EA+conventional, Escitalopram, Citalopram, Sertraline, Fluoxetine, and the control group in adverse events (Table 5).

**Table 5:** Results of pairwise meta-analyses for adverse events.

**Pairwise meta-analyses**								
**Comparative** **medications**	**Reference** **medications**	**Number of studies**	**Number of patients**	**RR**	**OR**	**95% CI**	**I^2^**	***P*-value**
Acupuncture/EA + conventional	conventional treatment	2	158	0.63		[0.21, 1.83]	38	*P* = 0.39
Acupuncture/EA (+placebo)	antidepressants (+sham acupuncture)	5	296	0.16		[0.07, 0.39]	35	*P* < 0.00001
Escitalopram	Control	7	996	1.31		[0.86, 1.99]	59	*P* = 0.21
Electroacupuncture	Antidepressants	9	859	0.21		[0.14, 0.32]	0	*P* < 0.00001
Modified sini san	Control	4	310		0.12	[0.06, 0.24]	37	*P* < 0.00001
Citalopram	Other SSRIs	7	510	0.85		[0.65, 1.10]	0	*P* = 0.21
Citalopram	SNRIs	4	282	0.95		[ 0.71, 1.26]	0	P=0.70
Sertraline	Control	11	4657	0.94		[0.83, 1.06]	45	*P* = 0.33
Chinese herbal medicine	Control	11	NA	0.49		[0.31, 0.77]	50	*P* = 0.03
Fluoxetine	Control	3	158		0.88	[0.31, 2.49]	0	*P* = 0.82

## Discussion

Our umbrella review was conducted by usingthe data derived from treatments of PSD patients, which was used to appraise the relative effectiveness of therapy. We attempted to summarize data from published meta-analyses and systematic reviews to indicate that there are significant beneficial treatment in patients with PSD.

The main theories of PSD are the neurotransmitter and cytokine hypotheses (Santos et al., 2009). Depression is associated with low levels of monoamines, especially 5-hydroxytryptamine, Norepinephrine, and dopamine (Krishnan and Nestler, 2008). Different symptoms of depression (cognition, emotion, and pain) may be related to the diverse neural systems. Changes in the limbic reward system of the dopaminergic midbrain may lead to the absence of delight hedonia (Nestler and Carlezon, 2006). Noradrenergic and serotonergic fibers originate from the brain stem nucleus and dominate the limbic system, prefrontal cortex, and related structures involved in emotion regulation. In addition, the descending pathway regulates pain through dorsolateral spinal protrusion. Furthermore, the cholinergic system has been considered related to the etiology of depression through nicotinic acetylcholine receptors. These pathways are associated with stroke lesions, leading to depression (Mineur and Picciotto, 2010). Antidepressants directly affect the brain or beyond their effects on depression, which may provide neuroprotection and promote brain neurogenesis (Loubinoux et al., 2012). Previous studies have indicated that SSRIs have beneficial effects on functional independence in PSD patients (Mead et al., 2012). Therefore, SSRIs were commonly used as a positive drug to ensure the sensitivity of the treatment for PSD.

It is speculated that low-frequency TMS stimulates inhibitory neurons while hfrTMS stimulates excitatory projection neurons, thus simulating neural plasticity through long-term enhancement (Duan et al., 2018). Therapy's benefits may be achieved by enhancing neuroplasticity, increasing the available concentration of key neurotransmitters, strengthening the positive emotional connection network, and reducing the connection in the negative emotional loops (Nordmann et al., 2015). Studies have shown that hfrTMS increases the concentration of BDNF, glucose metabolism in the cortex, neurogenesis, and regulation of neurobiochemical effects (Duan et al., 2018). Moreover, traditional Chinese medicine has the characteristics of multi-target and multi-pathway. Traditional Chinese medicine activates blood circulation, dissipates blood stasis, sedation, and hypnosis for patients with blood stasis syndrome, insomnia, and anxiety after stroke. It increases the secretion of neurotransmitters and 5-hydroxytryptophan and is widely used to treat PSD (Ding et al., 2021; Wan et al., 2021). Effective moxibustion is divided into four aspects: warming effect, medicinal penetration, infrared radiation, and aromatherapy (Huang et al., 2017). Long-term moxibustion can promote the'brain's uptake of L-tryptophan and shift L-tryptophan metabolism to 5-Hydroxytryptamine (Li et al., 2019). Previous studies have suggested that exercise is a complementary treatment for depression (De Man-Van Ginkel et al., 2010). Exercise may affect the hypothalamic-pituitary-adrenal (HPA) axis and immune function, improve the regulation of HPA response and increase immunity (Sigwalt et al., 2011). Cognitive-behavioral therapy can identify the emotional state of patients, overcome emotional disabilities, and treat depression and anxiety in PSD (Eum and Yim, 2015). Moreover, we considered the treatment with hfrTMS, Acupuncture/EA+conventional treatment, Escitalopram, Modified Sini San, Moxibustion, Xiaoyao Formula, Paroxetine, Chinese herbal medicine, Exercise, Citalopram, and Cognitive behavioral therapy to have a beneficial effect on PSD.

The safety of the treatments is critical in the treatment of PSD. The number of participants with at least one adverse event such as gastrointestinal symptoms, nausea, headache, insomnia, cardiovascular events, and other disorders was extracted. In addition, in the present review, the incidence of withdrawals as a result of adverse events with Acupuncture/EA(+placebo), Electroacupuncture, Modified Sini San, and Chinese herbal medicine treatments tended to be lower than control groups. Moreover, our study summarized that there were no significant differences between Acupuncture/EA+conventional, Escitalopram, Citalopram, Sertraline, Fluoxetine, and the control group in adverse events.

### Limitations

First, the comparative evidence of PSD treatments in this study was relatively small. Second, there may be other factors that may lead to this study's inconsistencies, such as the quality of the researched studies. Furthermore, a considerable number of researchers lack the abovementioned data, which makes less studies available.

## Conclusion

In conclusion, we found that 11 treatment methods may effectively improve the condition of PSD patients. In the future, patients with PSD should be treated with significant beneficial therapies, contributing to the successful construction of similar studies.

## Data availability statement

The original contributions presented in the study are included in the article/supplementary material, further inquiries can be directed to the corresponding authors.

## Author contributions

JX and XG collected the data. JX, TL, XF, and FF analyzed the data and prepared tables. JX wrote the manuscript. TL and SH designed the research. All authors contributed to the article and approved the submitted version.

## Funding

This study was supported by the experimental animal project of Zhejiang Basic Public Welfare Research Program (No. LGD20H090001), the Natural Science Foundation of Huzhou city (No.2018YZ03), the National Natural Science Foundation, China (No. 82004078), the Natural Science Foundation of Shandong Province (ZR2021LZY018).

## Conflict of interest

The authors declare that the research was conducted in the absence of any commercial or financial relationships that could be construed as a potential conflict of interest.

## Publisher's note

All claims expressed in this article are solely those of the authors and do not necessarily represent those of their affiliated organizations, or those of the publisher, the editors and the reviewers. Any product that may be evaluated in this article, or claim that may be made by its manufacturer, is not guaranteed or endorsed by the publisher.
